# Relationship between dietary factors and bisphenol a exposure: the second Korean National Environmental Health Survey (KoNEHS 2012–2014)

**DOI:** 10.1186/s40557-017-0200-1

**Published:** 2017-10-18

**Authors:** Jin-Soo Park, Seyoung Kim, Minkyu Park, Yeji Kim, Hyeeun Lee, Hyunrim Choi, Sinye Lim

**Affiliations:** 1 Department of Occupational and Environmental Medicine, Kyung Hee University Hospital, Seoul, South Korea; 2 Department of Occupational and Environmental Medicine, School of Medicine Kyung Hee University, Seoul, South Korea

**Keywords:** Bisphenol a, Dietary factors, Korean national environmental health survey

## Abstract

**Background:**

This study was aimed at finding out the exposure level of bisphenol A (BPA), a well-known endocrine disruptor, in relation to dietary factors using a data representing the Korean general population.

**Methods:**

This study was performed on 5402 adults aged 19 years and older based on the Second Korean National Environmental Health Survey (KoNEHS 2012–2014). The data analyzed urinary BPA concentration in relation to socio-demographic variables, health behavior-related variables, and dietary factor-related variables. Odds ratio (OR) was calculated through a logistic regression analysis after dividing the participants into high BPA exposure group and low BPA exposure group based on the top 75 percentile concentration. The logistic regression analysis was carried out considering the appropriate sample weight, stratification, and clustering of the second KoNEHS sample design.

**Results:**

The group drinking bottled water at home and the group using zip-top bags/plastic bags showed significantly higher urinary BPA concentration in female. OR tends to increase as the intake frequency of frozen food increased and OR of frozen food consumption of more than once a week was 1.48 (95% confidence interval (CI) 1.02–2.24) for male and the group drinking bottled water showed significantly higher OR of 1.45 (95% CI 1.06–2.17) after adjusting the related factors for female.

**Conclusions:**

BPA levels were high in female using bottled water and in male consuming frozen food, and therefore bottled water and frozen food need to be avoided to reduce BPA levels.

## Background

Bisphenol A (BPA) is synthesized by the condensation of two phenols with one acetone and used as a monomer [1]. After BPA was first synthesized in 1891, it has been in commercial use from early 1950s [2].

BPA is mainly used to make polycarbonate plastic, epoxy resins, and thermal paper [1]. Polycarbonate plastic is clear, shatter-resistant and used to make common consumer goods, such as water bottles, food storage containers while epoxy resin is used as coatings on the inside of cans to prevent rust and corrosion [3]. In these cases, BPA is released as free form and can be absorbed into the body [4].

BPA, a well-known endocrine disruptor, interacts with female hormone receptors and activates the female hormones, showing weak estrogenic effects [2]. Such effects have been confirmed in animal testing as well, with signs of lower sperm counts, uterotrophic effects, precocious puberty effects on female animals, and effects on male reproductive organ development observed [5]. In human, it is associated with behavior disorder, such as aggression and hyperactivity, drug addiction, recurrent miscarriages, diabetes, coronary heart diseases, and liver toxicity [3, 5, 6].

In a study describing estimated BPA daily intake based on urine BPA concentration data in 30 countries worldwide, the estimated BPA daily intake was especially high for adult population of Daejon, Korea in an areal analysis [7]. In another study describing urinary BPA concentrations in general population in seven Asian countries, the geometric mean (GM) of urinary BPA in Korean general population was the highest after creatinine adjustment [8]. Therefore, the level of BPA exposure in Korean adult population is considered to be higher than those in other countries.

Direct contact with products containing BPA is the main source of BPA exposure with the main exposure route being diet via food and drink [5, 7]. Because over 90% of BPA exposure is from diet, BPA body burden can be associated with dietary habits [5, 9]. In previous studies, other related factors of BPA exposure were age, gender, Body Mass Index (BMI), household income, and smoking [10–14].

Even though a nationwide biomonitoring of BPA exposure was conducted on the Korean general population, there is still not enough number of studies exploring the relation between BPA exposure level and dietary factors. Therefore, the purpose of this study was to analyze the relationship between urinary BPA concentration and dietary factors using data representing the Korean general population.

## Methods

### Study participants

This study was analyzed based on the Second Korean National Environmental Health Survey (KoNEHS 2012–2014). The second KoNEHS is designed to understand the Korean population’s exposure level of environmental pollutants and explore the influencing factors. The KoNEHS is conducted every 3 years by the National Institute of Environmental Research under the Ministry of Environment.

The second KoNEHS area consisted of 16 cities and provinces nationwide and for the survey sample size the enumeration district of the National Population and Housing Census 2010 was used as the population with the initial stratification classified by local administration. The secondary stratification was classified by socio-economic factors, using the stratified multistage cluster sampling applied with the proportional allocation of square root of population to extract 400 sample enumeration districts. Around 15 people from each sample enumeration district were surveyed targeting total of 6478 Koreans aged 19 years and older. The survey consists of an environmental exposure-related questionnaire, clinical tests, and analysis of environmental harmful substances in biological samples.

In this study, 5402 subjects (2488 men, 2914 women), excluding 928 subjects whose urinary creatinine concentration exceeded the proper range of 0.3–3.0 g/L and 215 subjects with missing urinary BPA concentration data, were included among the total participants of 6478 people. This study was approved by the Institutional Review Board (IRB) of Kyung Hee University Hospital (KHUH 2017–03-049). And this study only used the published data of the second KoNEHS.

### Variables

#### Urinary bisphenol A

Urinary BPA concentration was measured by Ultra Performance Liquid Chromatography - Mass Spectrometry (UPLC-MS) using spot urine. After hydrolyzing the urine sample with ß-glucuronidase/aryl sulfatase-degrading enzyme, the metabolites of BPA were extracted with ethyl ether and measured. The principle of analysis figures out the value of sample concentration using the calibration curve constructed by the Standard Addition Method which adds a fixed amount of standard solution to the sample [15]. The outcome of the calibration standard solution was set at reference value ±15% and the precision of reproducibility of the test method was indicated with a relative standard deviation of 15% while the QC (Quality Control) sample was used for measurement accuracy. QC standard solution was used for internal quality control and analyzed with precision and accuracy. The Method Detection Limit (MDL) of BPA metabolite was 0.15 μg/L. The value below MDL was substituted with the values with MDL divided by square root of 2 while the final BPA concentration was calculated after adjusting urinary creatinine concentration.

#### Dietary factors

The variables related to potential BPA exposure among the variables associated with dietary factors were reclassified in the following manner for this study. The type of drinking water at home was classified into filtered water, tap or boiled water, bottled water, and mineral or ground water while the frequency of canned food consumption and frozen food consumption were classified into rarely consumed, consumed less than once a week, and consumed more than once a week. Food containers used in refrigerator were classified into glass/metal/porcelain ware, plastic ware, and zip-top bags/plastic bags.

#### Potential confounders

The study participant’s socio-demographic variables and health behavior-related variables were classified in the following manner. Among the socio-demographic variables, the age was classified into units of 10 years from the age of 19. Body Mass Index (BMI) was classified into underweight for BMI of less than 18.5 kg/m^2^, normal weight for BMI of 18.5–25 kg/m^2^, and overweight for BMI of greater than or equal to 25 kg/m^2^, whereas the level of educational attainment was classified into less than high school, graduated from high school, and graduated from junior college or more. The marital status was classified into single, married, and others (divorced/death of spouse/separated) while the household income was classified into four groups according to the quartile. Alcohol consumption was classified into non-drinker for participants who did not drink at all or drank in the past but not anymore, light drinker for participants drinking less than heavy drinker, and heavy drinker for participants drinking 3 times and more a week and drinking more than 7 glasses per occasion for male (more than 5 glasses for female). The exercise status was classified into exercising group for participants exercising more than 3 times a week over 20 min and sweating during workout and non-exercising group for participants who do not fall under the exercising group. Smoking was classified into smoker for participants currently smoking and non-smoker for those who never smoked or quit smoking.

### Statistical analysis

The final data analysis of this study was analyzed by applying the weights presented in the original publicly open final dataset in accordance with the KoNEHS analysis guideline. The frequency and proportion of each variable were presented after gender stratification to figure out the general characteristics of the participants and chi-square tests were carried out to analyze the difference in the distribution of each variable. The geometric mean (GM) of urinary BPA was calculated by converting the natural logarithm because the urinary BPA level shows right deviation. Analysis of variance (ANOVA) was applied to compare the geometric mean value of metabolites in relation to dietary factors and Analysis of covariance (ANCOVA) to compare the geometric mean value with adjustments made to each variable. Odds ratio (OR) was calculated by using logistic regression by dividing the participants into high exposure group and low exposure group based on the top 75 percentile concentration (male: 2.37 μg/g creatinine, female: 3.15 μg/g creatinine). The logistic regression analysis was carried out considering the appropriate sample weight, stratification, and clustering of the second KoNEHS sample design. After gender stratification, in Model 1, no adjustments were made while age, BMI, socio-economic variables, health behavior-related variables and dietary factors were adjusted in Model 2. The IBM SPSS (version 19 for Windows) was used for the statistical analysis and the statistical significance level was set at *p* < 0.05.

## Results

Table 1 shows the characteristics configuration of the study participants analyzed in this study (Table 1). Male accounted for 2488 (46.1%) and female 2914 (53.9%). The GM of the urinary BPA concentration was 1.33 μg/g creatinine for male and 1.77 μg/g creatinine for female, with female showing significantly higher concentration (*p* < 0.001). For age, urinary BPA level increased with age for both male and female showing a decreasing tendency after the age of 50s for male and the age of 40s for female. For male, urinary BPA concentration increased significantly as alcohol consumption increased with the heavy drinker group showing the highest concentration of 1.58 μg/g creatinine. For female, a statistical significant difference was observed in relation to the educational attainment with the high school graduates showing the highest concentration of 1.91 μg/g creatinine, followed by junior college graduates or more, and junior high graduates. For household income, urinary BPA concentration increased significantly as the income increased with the highest income class showing the highest concentration of 2.05 μg/g creatinine in female.

**Table 1:** Demographic distributions of the study subjects and urinary concentration of bisphenol A (μg/g creatinine) according to general characteristics

Category		Total	Male	Female		Total			Male			Female		
		n (%)	n (%)	n (%)	*p* value*	GM (SE)	95% CI	*p* value	GM (SE)	95% CI	p value	GM (SE)	95% CI	*p* value
Total		5402 (100)	2488 (46.1)	2914 (53.9)		1.55 (1.01)	1.52–1.60		1.33 (1.02)	1.29–1.38		1.77 (1.02)	1.71–1.83	<0.001
Age	19–29	467 (8.6)	235 (9.4)	232 (8.0)	0.032	1.55 (1.05)	1.42–1.69	<0.001	1.34 (1.07)	1.18–1.52	0.021	1.79 (1.06)	1.59–2.02	0.003
(years)	30–39	909 (16.8)	400 (16.1)	509 (17.5)		1.57 (1.03)	1.49–1.70		1.34 (1.05)	1.23–1.46		1.79 (1.04)	1.65–1.94	
	40–49	1046 (19.4)	473 (19.0)	573 (19.7)		1.63 (1.03)	1.53–1.72		1.37 (1.05)	1.25–1.51		1.89 (1.04)	1.76–2.04	
	50–59	1207 (22.3)	524 (21.1)	683 (23.4)		1.67 (1.03)	1.58–1.77		1.47 (1.04)	1.35–1.59		1.85 (1.04)	1.72–1.98	
	60–69	1082 (20.0)	523 (21.0)	559 (19.2)		1.50 (1.03)	1.42–1.59		1.30 (1.04)	1.19–1.41		1.72 (1.04)	1.59–1.86	
	≥70	691 (12.8)	333 (13.4)	358 (12.3)		1.31 (1.04)	1.22–1.41		1.15 (1.05)	1.01–1.28		1.48 (1.05)	1.05–1.63	
BMI	<18.5	139 (2.6)	45 (1.8)	94 (3.2)	<0.001	1.56 (1.08)	1.33–1.83	0.949	1.39 (1.16)	1.04–1.84	0.530	1.65 (1.10)	1.36–1.99	0.744
(kg/m^2^)	18.5–24.9	3138 (58.1)	1393 (56.0)	1745 (59.9)		1.55 (1.02)	1.50–1.60		1.31 (1.03)	1.24–1.38		1.77 (1.02)	1.70–1.85	
	≥25.0	2125 (39.3)	1050 (42.2)	1075 (36.9)		1.56 (1.02)	1.50–1.63		1.37 (1.03)	1.29–.1.45		1.78 (1.03)	1.68–1.88	
Education	≤ Middle school	1811 (33.5)	673 (27.0)	1138 (39.1)	<0.001	1.52 (1.02)	1.45–1.59	0.171	1.33 (1.04)	1.24–1.43	0.904	1.64 (1.03)	1.56–1.73	0.001
	High school	1655 (30.6)	764 (30.7)	891 (30.6)		1.61 (1.02)	1.54–1.69		1.32 (1.04)	1.23–1.41		1.91 (1.03)	1.80–2.03	
	≥ College	1936 (35.8)	1051 (42.2)	885 (30.4)		1.54 (1.02)	1.48–1.61		1.35 (1.03)	1.27–1.43		1.81 (1.03)	1.70–1.92	
Marital	Single	568 (10.5)	319 (12.8)	249 (8.5)	<0.001	1.48 (1.04)	1.37–1.60	0.351	1.36 (1.06)	1.22–1.51	0.784	1.66 (1.06)	1.48–1.86	0.101
status	Married	4302 (79.6)	2053 (82.5)	2249 (77.2)		1.57 (1.01)	1.53–1.62		1.33 (1.02)	1.27–1.38		1.83 (1.02)	1.76–1.90	
	Others	532 (9.8)	116 (4.7)	416 (14.3)		1.50 (1.04)	1.38–1.62		1.40 (1.09)	1.18–.1.68		1.52 (1.05)	1.39–1.66	
Household	1st quartile	1489 (27.6)	695 (27.9)	794 (27.2)	0.355	1.47 (1.03)	1.40–1.55	0.002	1.27 (1.04)	1.18–1.36	0.258	1.68 (1.03)	1.57–1.79	0.016
income	2nd quartile	2510 (46.5)	1168 (46.9)	1342 (46.1)		1.54 (1.02)	1.48–1.59		1.34 (1.03)	1.26–1.41		1.74 (1.03)	1.65–1.82	
	3rd quartile	1352 (25.0)	598 (24.0)	754 (25.9)		1.68 (1.03)	1.59–1.77		1.41 (1.04)	1.30–1.52		1.93 (1.03)	1.81–2.06	
	4th quartile	51 (0.9)	27 (1.1)	24 (0.8)		1.72 (1.14)	1.32–2.24		1.47 (1.21)	1.02–2.12		2.05 (1.21)	1.42–2.98	
Smoking	None or Ex-smoker	4380 (81.1)	1579 (63.5)	2801 (96.1)	<0.001	1.61 (1.01)	1.56–1.65	<0.001	1.35 (1.02)	1.29–1.41	0.467	1.77 (1.02)	1.71–1.83	0.705
	Current smoker	1022 (18.9)	909 (36.5)	113 (3.9)		1.35 (1.03)	1.27–1.43		1.31 (1.03)	1.23–1.39		1.71 (1.09)	1.44–2.03	
Alcohol	None	2163 (40.0)	709 (28.5)	1454 (49.9)	<0.001	1.56 (1.02)	1.50–1.63	0.282	1.26 (1.04)	1.17–1.35	0.001	1.74 (1.02)	1.66–1.82	0.326
	Light drinker^a^	2762 (51.1)	1393 (56.0)	1369 (47.0)		1.53 (1.02)	1.48–1.59		1.31 (1.03)	1.25–1.38		1.79 (1.03)	1.71–1.88	
	Heavy drinker^b^	477 (8.8)	386 (15.5)	91 (3.1)		1.65 (1.04)	1.51–1.80		1.58 (1.05)	1.43–1.74		1.98 (1.10)	1.64–2.40	
Exercise	No	3918 (72.5)	1743 (70.1)	2175 (74.6)	<0.001	1.55 (1.02)	1.50–1.60	0.683	1.33 (1.02)	1.27–1.39	0.826	1.75 (1.02)	1.68–1.82	0.246
	Yes^c^	1484 (27.5)	745 (29.9)	739 (25.4)		1.57 (1.03)	1.49–1.65		1.34 (1.04)	1.25–1.44		1.83 (1.03)	1.71–1.96	

*p value calculated by chi-square tests

*GM* geometric mean/*SE* standard error/*CI* confidence Interval

Light drinker^a^: drinking less than heavy drinker/Heavy drinker^b^: drinking ≥3 times a week and ≥7 glasses per occasion for male (≥5 glasses for female)

Exercise^c^: exercising ≥3 times a week over 20 min and sweating during workout

Urinary BPA concentration according to dietary factors after gender stratification was presented in Table 2 before and after adjustments were made to the related factors (Table 2). For male, participants drinking bottled water showed the highest concentration of 1.46 μg/g creatinine. The concentration increased as the intake frequency of canned food and frozen food increased. Furthermore, users of zip-top/plastic bags showed the highest concentration without statistical significance in male. For female, participants drinking bottled water showed the highest concentration of 1.87 μg/g creatinine (*p* = 0.011). The concentration increased as the intake frequency of canned food and frozen food increased. In addition, users of zip-top/plastic bags showed the highest concentration of 1.81 μg/g creatinine (*p* = 0.036).

**Table 2:** Urinary geometric mean (GM) concentration of bisphenol A (*μ*g/g creatinine) according to variable categorized by dietary factors

	Male							Female						
	Crude				Adjusted^a^			Crude				Adjusted^a^		
Category	n (%)	GM (SE)	95% CI	*p* value	GM (SE)	95% CI	*p* value	n (%)	GM (SE)	95% CI	*p* value	GM (SE)	95% CI	*p* value
At-home drinking water														
Filtered water	972 (39.1)	1.30 (1.03)	1.22–1.38	0.141	1.29 (1.03)	1.21–1.37	0.102^a^	1180 (40.5)	1.78 (1.03)	1.71–1.85	0.037	1.81 (1.03)	1.74–1.88	0.011*
Tap or boiled water	907 (36.5)	1.30 (1.03)	1.23–1.40		1.31 (1.03)	1.22–1.39		1172 (40.2)	1.67 (1.03)	1.60–1.74		1.68 (1.03)	1.63–1.77	
Bottled water	309 (12.4)	1.48 (1.06)	1.29–1.66		1.46 (1.06)	1.28–1.63		285 (9.8)	1.89 (1.06)	1.70–2.07		1.87 (1.06)	1.68–2.04	
Mineral or ground water	300 (12.1)	1.40 (1.06)	1.21–1.59		1.43 (1.06)	1.24–1.60		277 (9.5)	1.85 (1.06)	1.66–2.04		1.83 (1.06)	1.64–2.01	
Canned food intake														
Rarely	1235 (49.6)	1.30 (1.02)	1.23–1.37	0.433	1.31 (1.02)	1.24–1.39	0.082^b^	1482 (50.9)	1.72 (1.02)	1.64–1.80	0.130	1.75 (1.02)	1.67–1.84	0.068^†^
< Once a week	846 (34.0)	1.36 (1.03)	1.28–1.46		1.33 (1.03)	1.25–1.45		1006 (34.5)	1.81 (1.03)	1.71–1.92		1.78 (1.03)	1.72–1.93	
≥ Once a week	407 (16.4)	1.37 (1.05)	1.25–1.51		1.36 (1.05)	1.23–1.49		426 (14.6)	1.86 (1.05)	1.70–2.03		1.80 (1.05)	1.64–1.97	
Frozen food intake														
Rarely	1991 (80.0)	1.31 (1.02)	1.26–1.37	0.048	1.32 (1.02)	1.26–1.37	0.055^c^	2343 (80.4)	1.75 (1.02)	1.68–1.82	0.290	1.76 (1.02)	1.69–1.82	0.165^‡^
< Once a week	334 (13.4)	1.37 (1.05)	1.23–1.52		1.35 (1.06)	1.13–1.53		406 (13.9)	1.85 (1.05)	1.69–2.02		1.77 (1.05)	1.61–1.95	
≥ Once a week	163 (6.6)	1.57 (1.08)	1.35–1.82		1.53 (1.08)	1.33–1.82		165 (5.7)	1.86 (1.08)	1.62–2.14		1.81 (1.08)	1.57–2.10	
Food container in refrigerator														
Glass or metal or porcelain ware	1096 (44.1)	1.34 (1.03)	1.26–1.42	0.483	1.33 (1.03)	1.26–1.42	0.159^d^	1338 (45.9)	1.49 (1.03)	1.41–1.56	0.060	1.49 (1.03)	1.41–1.57	0.036^§^
Plastic ware	1283 (51.6)	1.32 (1.03)	1.25–1.39		1.32 (1.03)	1.25–1.39		1445 (49.6)	1.75 (1.02)	1.69–1.80		1.76 (1.02)	1.70–1.81	
Zip-top or plastic bag	109 (4.4)	1.48 (1.10)	1.23–1.78		1.48 (1.10)	1.23–1.78		131 (4.5)	1.82 (1.08)	1.52–2.11		1.81 (1.08)	1.50–2.10	

*GM* geometric mean

*SE* Standard error

*CI* Confidence Interval

*Adjusted by age, alcohol, canned food intake, frozen food intake, and food container in refrigerator

^†^Adjusted by age, alcohol, at-home drinking water, frozen food intake, and food container in refrigerator

^‡^Adjusted by age, alcohol, at-home drinking water, canned food intake, and food container in refrigerator

^§^Adjusted by age, alcohol, at-home drinking water, canned food intake, and frozen food intake

The logistic regression was conducted based on the top 75 percentile BPA concentration in Table 3. OR of participants consuming frozen food more than a week was significant in male at 1.48 (95% confidence interval (CI) 1.02–2.24) in Model 2. OR of participants drinking bottled water was significant in female at 1.45 (95% CI 1.06–2.17) in Model 2. But for canned food consumption and food container in refrigerator, ORs were not statistically significant.

**Table 3:** Odds ratios (OR) and 95% confidence intervals (CI) relating dietary factors of high urinary concentration of bisphenol A (male: ≥ 2.37 *μ*g/g creatinine, female: ≥3.15 *μ*g/g creatinine) compared to low urinary concentration of bisphenol A

Category	Male		Female	
	Model 1^a^	Model 2^b^	Model 1^a^	Model 2^b^
At-home drinking water				
Filtered water	1	1	1	1
Tap water or boiled water	1.09(0.84–1.42)	1.11(0.85–1.45)	0.91(0.72–1.15)	0.95(0.75–1.20)
Bottled water	1.24(0.84–1.82)	1.23(0.83–1.81)	1.40(1.03–2.10)	1.45(1.06–2.17)
Mineral or ground water	1.05(0.69–1.59)	1.04(0.68–1.59)	0.92(0.63–1.36)	0.96(0.65–1.43)
Canned food intake				
Rarely	1	1	1	1
< Once a week	0.95(0.72–1.25)	0.94(0.67–1.32)	1.15(0.92–1.45)	1.11(0.87–1.42)
≥ Once a week	1.06(0.78–1.44)	1.00(0.68–1.48)	1.18(0.90–1.56)	1.17(0.85–1.60)
Frozen food intake				
Rarely	1	1	1	1
< Once a week	1.10(0.78–1.58)	1.12(0.81–1.61)	1.05(0.69–1.61)	1.11(0.70–1.76)
≥ Once a week	1.42(0.98–2.12)	1.48(1.02–2.24)	1.18(0.73–1.91)	1.20(0.73–1.95)
Food container in refrigerator				
Glass or metal or porcelain ware	1	1	1	1
Plastic ware	1.06(0.57–1.97)	1.10(0.61–2.01)	1.20(0.77–1.89)	1.15(0.73–1.81)
Zip-top or plastic bag	1.06(0.57–1.96)	1.12(0.62–2.02)	1.22(0.75–2.01)	1.20(0.73–1.97)

^a^Model 1: crude model

^b^Model 2: age, BMI, sociodemographic status (education, marital status, and household income), individual factors (alcohol intake, exercise, and smoking), and dietary factors

## Discussion

We analyzed the relationship between urinary BPA concentration and dietary factors using national survey data. The main finding of the present study is that high BPA concentration was associated with the use of bottled water in women and frozen food intake in men.

It was confirmed that BPA can leach out of plastic beverage bottles, and exposure to BPA increased when these beverages were consumed [1]. Adult group drinking bottled water showed significantly higher urinary BPA concentrations compared to those who did not drink bottled water, and a study found that urinary BPA concentrations were higher for drinking bottled water than boiled tap water, and drinking water using polycarbonate bottle than ceramic cup [11, 16]. In an intervention study, the concentration was 1.2 μg/g creatinine when measured after not using the bottle for a week and the concentration increased by around 69% to 2.0 μg/g creatinine after using the bottle for a week [17]. These studies are believed to have something in common with the outcome of the present study showing the highest OR in female group drinking bottled water. In the case of men, the group drinking bottled water did not show a statistical significance in the outcome as it is assumed that men drink less amount of water at home due to their higher labor force participation rate in comparison to women [18].

Frozen food intake and BPA exposure can be associated with packaging material for frozen food, cooking method and food container. Plastic wraps used in food packaging can cause BPA migration, and urinary BPA concentrations decreased when consumption of plastic packaged food were limited for 3 days compared to when on a general diet [19, 20]. It can be presumed through these studies that the use of plastic packaging for most frozen foods is one of the causes of increased BPA exposure. Also, BPA migration can occur from plastic containers used for microwave oven, and BPA exposure could increase when using vinyl wrapping in a microwave oven to defrost frozen food [21, 22]. The present study shows that the geometrical mean of urinary BPA levels was significant in female group using zip-top or plastic bags as refrigerator storage containers(*p* = 0.036). Therefore, use of these containers for frozen food storage can also increase BPA exposure as a result of frozen food consumption.

In many studies, BPA was detected in commercial canned foods [23, 24]. And the results of the analysis carried out on BPA concentration in Korean canned food showed that the average concentration was highest for coffee at 45.51 mg/kg, followed by tuna, meat, fruit, tea, and vegetable [25]. Also, the amount of BPA detected in canned food was over 100 times more than BPA found in non-canned food or food packaged in glass, plastic paper and laminate paperboard, while a study calculating the dietary intake of BPA in urine for a day confirmed that intake of BPA mostly came from canned food [7, 26]. Although not statistically significant in the present study, these studies explained that urinary BPA concentrations tend to rise as the number of canned food consumption increased. In the Stage 1 survey of KoNEHS, the GM of the urinary BPA concentration of total study participants was 0.884 μg/g creatinine and 1.48 μg/g creatinine in the Stage 2 survey. The increased consumption rate of canned food from 42.9% to 48.7% was suggested as one of contributing factors causing the Stage 2 survey results to be higher compared to that of Stage 1 survey results. In a Japanese study conducted on university students, urinary BPA level for frequent drinking of tea or beverages can was significantly high in 1992. However, such tendency was not observed in 1999 on the basis that majority of beverage companies changed to can coatings which did not contain BPA in 1997 [23]. Therefore, to reduce BPA exposure, the use of an alternate product which hardly releases BPA may be effective.

Although the present study analyzed the level of BPA exposure from food container in refrigerator, there was no statistical significance in OR. The reason for that is that BPA is included in polycarbonate plastic or polyvinyl chloride plastic used in food storage products, and that migration levels of BPA from containers vary for foods with different pH [1, 24]. Furthermore, according to a Korean study researching the validity of the survey on containers used mostly for food storage, the exact agreement of survey responses was only around 72%, and although the KoNEHS survey question used for the present study chose only one type of container, there is the possibility that each household may be using many types of storage containers simultaneously [27].

In the study of temporal trends in BPA exposure conducted in NHANES (2003–2012), female, daily smoker, and low household income group showed a significantly higher concentration [28]. Similar to this study, the present study showed a significant difference of urinary BPA concentration in gender, age, household income, and smoking in total participants.

The limitations of this study are as follows. Firstly, the impact of dietary factors outside the home on BPA concentration could not be determined as only dietary factors were included in the analysis. Secondly, a more detailed content analysis on the dietary factors, such as the effect of amount of daily water intake, types of canned food and frozen food, and storage period, and type of food stored in the food containers for refrigerator could not be performed. To resolve this problem, an in-depth analysis needs to be carried out by adding detailed questionnaire later on based on the study performed abroad on exposure factors. Thirdly, only the data on BPA concentration measured in random spot urine was analyzed in this study. The sampling method needs improvement, such as multiple sampling or 24-h urine collection because of BPA’s short half-life and variation in a day, and an analysis needs to be performed not only on urine samples but also on blood samples [3, 29].

Despite these limitations, this study explored BPA exposure levels of Korean adults using the KoNEHS representing the Korean general population and found out the association between urinary BPA and dietary factors.

## Conclusions

BPA levels were high in female using bottled water and in male consuming frozen food. According the result of the present study, bottled water and frozen food need to be avoided to reduce BPA levels. Further research should be needed to investigate detailed dietary factors associated with BPA concentration.

## Abbreviations

ANCOVA: Analysis of covariance
ANOVA: Analysis of variance
BMI: Body Mass Index
BPA: Bisphenol A
CI: Confidence Interval
IRB: Institutional Review Board
KoNEHS: Korean National Environmental Health Survey
MDL: Method Detection Limit
NHANE: National Health and Nutrition Examination Survey
OR: Odds Ratio
QC: Quality Control
UPLC-MS: Ultra Performance Liquid Chromatography - Mass Spectrometry
